# Necrology

**Published:** 1902-06

**Authors:** 


### NECROLOGY.

At Factoryville, on May 9th, Dr. D. C. Stanton, veterinary surgeon, died after a long period of illness. Dr. Stanton was a member of the Pennsylvania State Veterinary Medical Association and a regular attendant at its conventions. He took an active intereest in all its work and affairs.

Charles Scheile, veterinary surgeon, of Quincy, Illinois, died recently.

B. S. Sutton, veterinary surgeon, of Carpenteria, California, died recently.

